# Biomolecular computers with multiple restriction enzymes

**DOI:** 10.1590/1678-4685-GMB-2016-0132

**Published:** 2017-10-23

**Authors:** Sebastian Sakowski, Tadeusz Krasinski, Jacek Waldmajer, Joanna Sarnik, Janusz Blasiak, Tomasz Poplawski

**Affiliations:** 1 Faculty of Mathematics and Computer Science, University of Lodz, Lodz, Poland; 2 Logic, Language and Information Group, Department of Philosophy, University of Opole, Opole, Poland; 3 Department of Molecular Genetics, University of Lodz, Lodz, Poland

**Keywords:** bioinformatics, DNA, DNA computer, restriction enzymes

## Abstract

The development of conventional, silicon-based computers has several limitations, including some related to the Heisenberg uncertainty principle and the von Neumann “bottleneck”. Biomolecular computers based on DNA and proteins are largely free of these disadvantages and, along with quantum computers, are reasonable alternatives to their conventional counterparts in some applications. The idea of a DNA computer proposed by Ehud Shapiro’s group at the Weizmann Institute of Science was developed using one restriction enzyme as hardware and DNA fragments (the transition molecules) as software and input/output signals. This computer represented a two-state two-symbol finite automaton that was subsequently extended by using two restriction enzymes. In this paper, we propose the idea of a multistate biomolecular computer with multiple commercially available restriction enzymes as hardware. Additionally, an algorithmic method for the construction of transition molecules in the DNA computer based on the use of multiple restriction enzymes is presented. We use this method to construct multistate, biomolecular, nondeterministic finite automata with four commercially available restriction enzymes as hardware. We also describe an experimental applicaton of this theoretical model to a biomolecular finite automaton made of four endonucleases.

## Introduction

**Figure 1:**
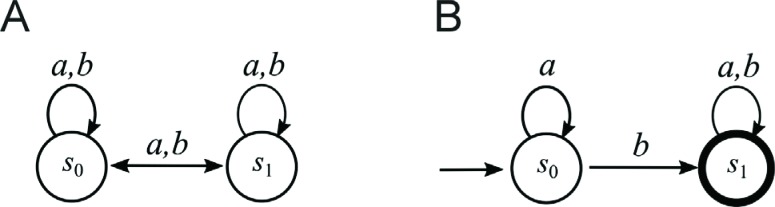
Finite automata with two states. (A) All possible eight transition rules for a two-state, two-symbol nondeterministic finite automaton. Computer programming involved the selection of some of these transition rules for the initial and final (accepted) states. (B) Graph representing an example of a two-state, two-symbol finite automaton *A*_1_ that accepts words with at least one symbol “*b*”.

Biomolecular computers are the answer to problems associated with the development of traditional, silicon-based computers, particularly their miniaturization, as implied by the Heisenberg uncertainty principle, and to limitations in data transfer to and from the main memory by the central processing unit (Amos, 2005). The first attempt to develop a DNA computer was by Adleman (1994), who solved some computational problems in a laboratory test-tube. Over the next two decades, numerous reports on DNA computing appeared. Some studies have focused on selected, well-known problems in mathematics and computer science, e.g., the tic-tac-toe algorithm (Stojanovic and Stefanovic, 2003), the Knight problem (Faulhammer *et al.*, 1999) or the SAT problem (Lipton, 1995). Other areas of research have attempted to apply DNA computing in medicine, e.g., for cancer therapy (Benenson *et al.,* 2004) or ‘miRNA’ level diagnostics (Seelig *et al.*, 2006). An interesting trend in DNA computing has been the development of biomolecular solutions for well-known models in theoretical computer science, such as finite automata, pushdown automata or Turing machines. Although some of this research provided only theoretical solutions without practical laboratory implementation, e.g., biomolecular representations of the Turing machine (Rothemund, 1995) or the pushdown automaton (Cavaliere *et al.*, 2005; Krasinski *et al.,* 2012), there have been prominent exceptions, including a stochastic automaton (Adar *et al.*, 2004) and a finite automaton (Benenson *et al.*, 2001, 2003). These first constructions of DNA computers used one restriction enzyme (RE) as the hardware and DNA fragments as the software and input/output signals. From a biochemical point of view, the DNA computer works by sequentially cutting and joining DNA molecules with the RE *Fok*I and DNA ligase. These DNA computers represent a class of devices known as nondeterministic finite automata that can solve simple computational problems. Benenson *et al.* (2001) designed and implemented a model of a two-state two-symbol (Figure 1A) nondeterministic finite state automaton – the simplest model of a computer (Hopcroft *et al.*, 2001).

Conventionally, finite automata (finite state machines) are used as controllers for electromechanical devices such as automatic doors and supermarket entrances (Sipser, 2006), as well as for many household devices such as dishwashers, electronic thermostats, digital watches and calculators. They can also be used as probabilistic tools to predict financial market prices and to recognize patterns in data analysis. Finite automata consist of a control unit equipped with a reading head and an input tape that, in a finite state, can read input words built of symbols from a finite set (called alphabet). The software of a finite automaton consists of transition rules that determine the sequence of states during computation. In each step, the automaton reads one symbol to the right of the input word and then changes its state according to the current transition rule. The input word is accepted if the automaton is in one of the final states after reading the whole word. Finite automata are generally represented in the form of graphs that allow one to display the relationship between objects (Sipser, 2006). Figure 1B shows an example of a two-state two-symbol finite automaton *A*_1_ in the form of a graph. The state diagram has two states labeled *s*_0_ and *s*_1_. The initial (starting) state is *s*_0_ – indicated by an arrow pointing to it from nowhere. The accepted state is *s*_1_ and is denoted with a thick circle. The arrows referred to as transitions show the relationship between states. When an automaton receives an input string (input word) such as *aabab*, it first processes this string and then produces an output to accept or reject. For example, the input word *aabab* can be processed by automaton *A*_1_ as follows: 1) Start action in state *s*_0_. 2) Read first symbol *a* from input word and move from state *s*_0_ to *s*_0_. 3) Read second symbol *a* from input word and move from state *s*_0_ to *s*_0_. 4) Read symbol *b* and move from state *s*_0_ to *s*_1_. 5) Read symbol *a* from input word and move from state *s*_1_ to *s*_1_. 6) Read symbol *b* from input word and move from state *s*_1_ to *s*_1_, and finally, 7) Accept input string because automaton *A*_1_ has read the whole input string *aabab* and is in accepted state *s*_1_.

**Figure 2:**
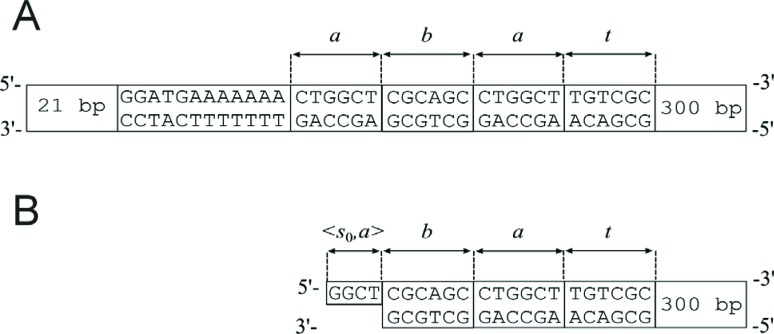
An example of an input molecule representing the word *aba*. The symbol *t* in the input word allows the detection of the final product of computation. (A) and (B) Before and after the first cut with endonuclease *Fok*I, respectively. bp – base pairs.

The biomolecular finite state machine proposed by Benenson *et al.* (2001) implemented the above scheme of computation using molecules and DNA processing proteins. The laboratory implementation of this DNA-based computer included one restriction enzyme (*Fok*I), DNA oligonucleotides as transition molecules, input signals and T4 DNA ligase. The restriction enzyme *Fok*I recognized the GGATG sequence (all DNA sequences are presented in the 5′ → 3′ direction, unless stated otherwise) and made an asymmetrical cut in double-stranded DNA. The automaton’s two symbols (*a* and *b*) and terminator *t* that signals the end of the word were coded by double-stranded DNA molecules of six base pairs in length (Figure 2A). Each of the input molecules had a *Fok*I recognition site and represented an input word consisting of the symbols *a* and *b*. They also contained flanking sequences to bind the enzyme and to detect the final state of computation. Single stranded overhangs produced by *Fok*I in the input molecule represented not only a symbol, but also a state of the machine (Figure 2B) (Benenson *et al.*, 2001).

**Figure 3:**
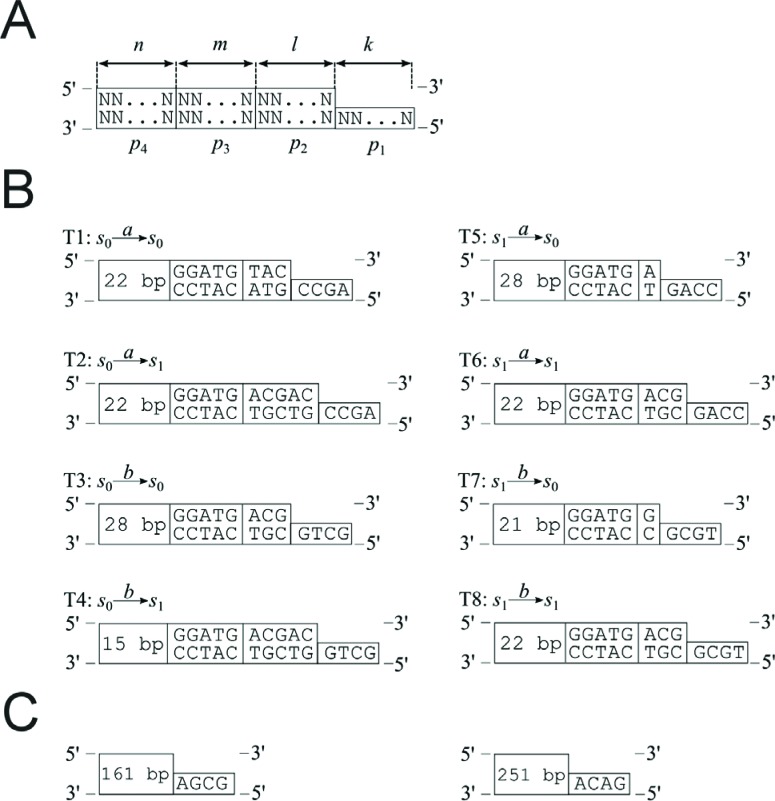
Transition molecules. (A) The parts of transition molecules. N indicates nitrogenous bases: *A* (adenine), *T* (thymine), *G* (guanine) and *C* (cytosine). (B) All possible transition rules and transition molecules in the two-state, two-symbol biomolecular automaton presented by Benenson *et al.* (2001). (C) Construction of the detection molecules for states *s*_0_ and *s*_1_.

The software (transition rules) was coded by DNA transition molecules (Figure 3B), containing the *Fok*I recognition sequence, spacers and sticky ends of a length characteristic for *Fok*I. Each of the transition molecules consisted of four parts that were DNA sequences made of nucleotides identified as *p*_1_, *p*_2_, *p*_3_ and *p*_4_ (Figure 3A). Part *p*_1_ of a transition molecule was single-stranded DNA while parts *p*_2_, *p*_3_ and *p*_4_ were double-stranded DNA (Figure 3A,B). Each part of the transition molecule was encoded by a different sequence of nucleotides and had a characteristic length: *k* for *p*_1_, *l* for *p*_2_, *m* for *p*_3_ and *n* for *p*_4_. The first part, *p*_1_ (a sticky end) of a transition molecule, was complementary to the single-stranded part of an input molecule and represented a pair < *state*, *symbol* > in a biomolecular finite automaton. The second part, *p*_2_ (a spacer part), allowed control of the depth of cutting into the input molecule. The third part, *p*_3_ (a restriction site), contained the sequence of nucleotides specific for a particular endonuclease and enabled this restriction enzyme to act. The last part, *p*_4_ (an additional part), aided ligation of the restriction enzyme to the whole DNA molecule because long DNA molecules are cut better by restriction enzymes. Parts *p*_1_, *p*_2_ and *p*_4_ did not contain the restriction enzyme cleavage site present in part *p*_3_.

In the biomolecular computer described by Benenson *et al.* (2001) there were additional elements (detection molecules) that, in laboratory experiments, recognized the final state of computation. These molecules consisted of sticky ends (AGCG and ACAG, representing the chosen final states *s*_0_ and *s*_1_, respectively) and an additional double-stranded fragment of DNA (the total length in each case being 161 bp and 251bp, respectively) (Figure 3C).

**Figure 4:**
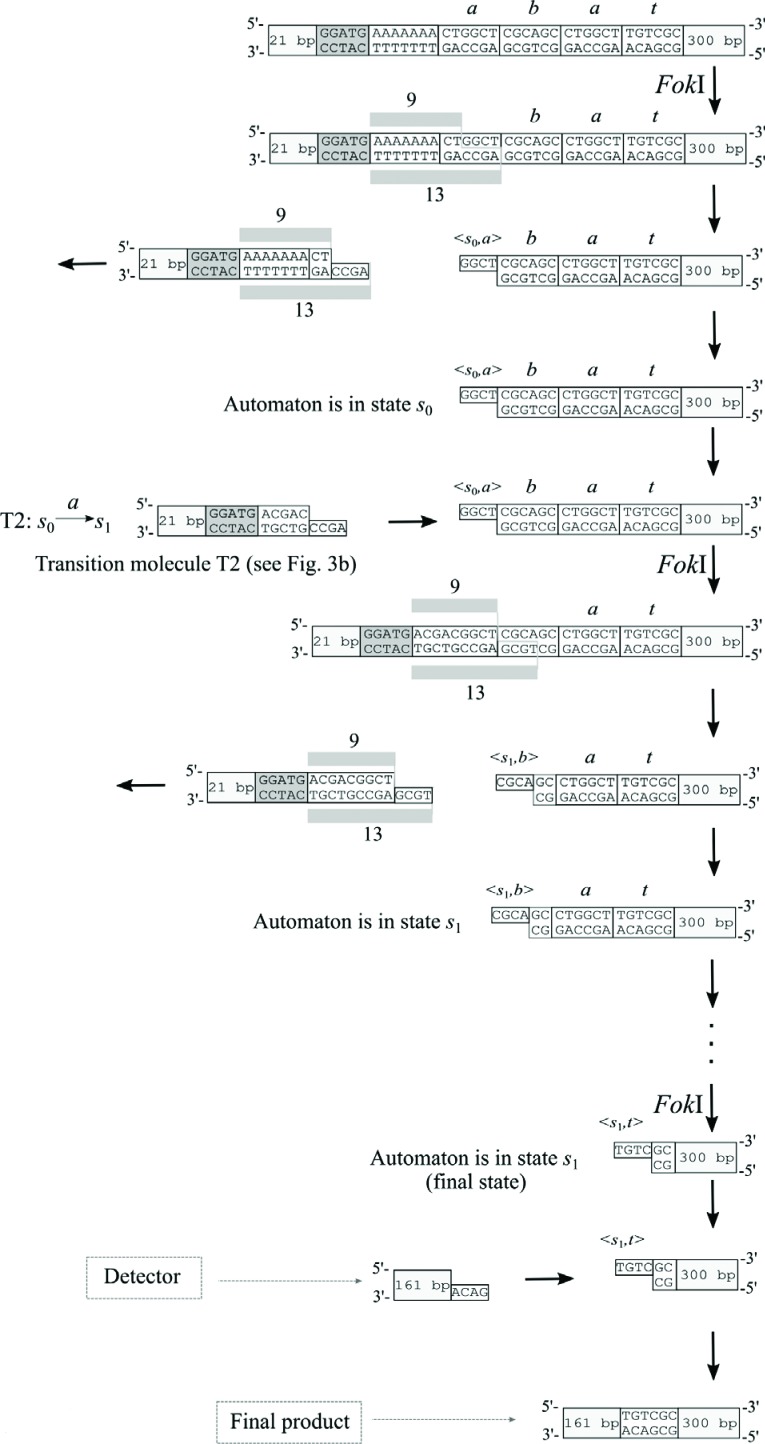
Transitions of a biomolecular automaton obtained using one endonuclease *Fok*I.

The finite automaton described above was produced in the laboratory by incubating *Fok*I, transition molecules, detection molecules and input molecules in a single tube. The computation process was initiated by cutting an input molecule with *Fok*I. In each cycle of the computation process, a transition molecule combined with the sticky ends of an input molecule followed by the sealing of two phosphodiester bonds by DNA ligase. *Fok*I could then cut within the next symbol and produce a sticky end representing a new < state, symbol > pair (Figure 4). This biomolecular computer was limited to two states and used only one restriction enzyme (*Fok*I). Since the initial description, other modifications have been incorporated into DNA-based computers (Unold *et al.*, 2004; Soreni *et al.*, 2005; Chen *et al.,* 2007) to improve their potential in biomedical sciences (Benenson *et al.*, 2004; Seelig *et al.*, 2006), including the use of two restriction enzymes (Krasinski and Sakowski, 2008; Krasinski *et al.*, 2013).

**Figure 5:**
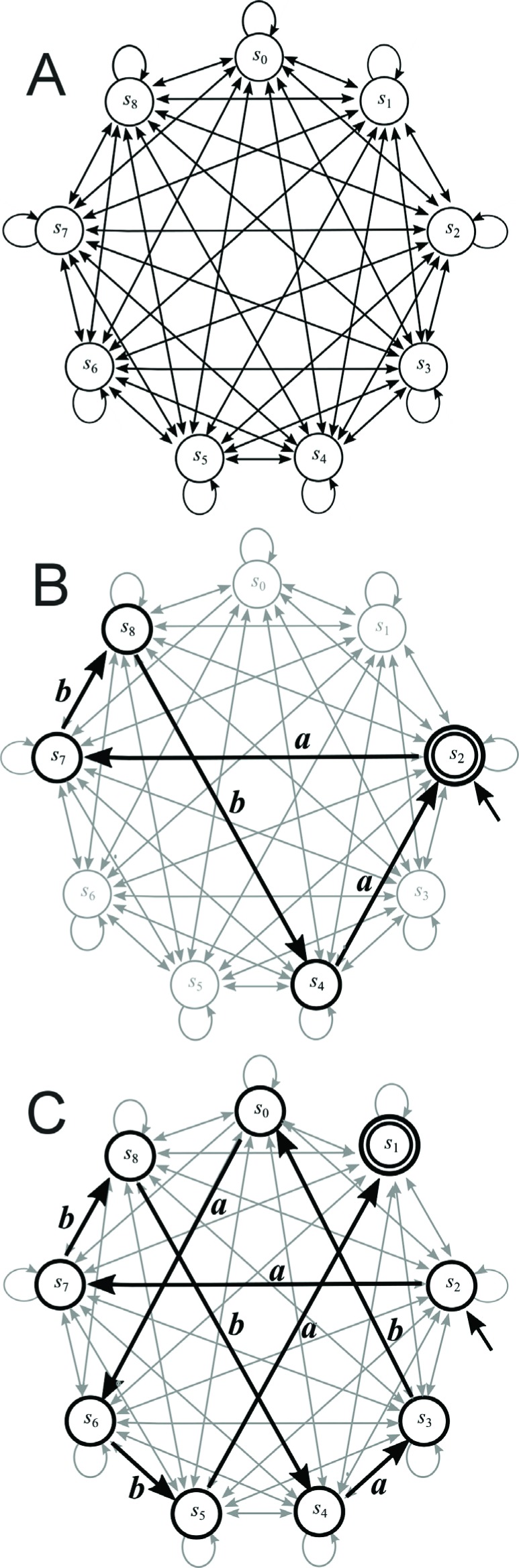
Finite automata with nine states. (A) All possible 162 transition rules for a nine-state, two-symbol nondeterministic finite automaton. On each arrow the symbols *a* and *b* should be placed. These 162 transition rules are coded by DNA molecules – see all 162 transition molecules in Tables S1-S8. (B) Graph showing an example of a four-state, two-symbol finite automaton *M*_1_. State *s*_2_ corresponds simultaneously to the initial and final states. This four-state automaton requires the autonomous action of four restriction enzymes and alternative splicing by the restriction enzymes *Bae*I, *Bbv*I, *Acu*I and *Mbo*I. (C) An example of a nine-state automaton (*s*_2_ – initial state, *s*_1_ – final state).

Based on these reports, we hypothesized that the number of states in a DNA-based computer could be extended by increasing the number of restriction enzymes. To assess this hypothesis, a set of appropriate transition molecules would need to be constructed. In this study, we developed all transition rules for 162 transition molecules (Supplementary material Tables S1-S8) in a biomolecular nine-state, two-symbol nondeterministic finite automaton *M* (Figure 5A) with four restriction endonucleases. We describe the results for the laboratory implementation of a biomolecular automaton involving four endonucleases (*Bae*I, *Bbv*I, *Acu*I and *Mbo*II). While preparing this model, we noted that the construction of transition molecules was relatively difficult and required an appropriate method to rapidly encode the particular transition molecules. We also present an algorithm for the construction of transition molecules in biomolecular automata with multiple restriction enzymes. This algorithm was used to construct a multistate biomolecular nondeterministic finite automaton with multiple commercially available restriction enzymes as hardware.

## Materials and Methods

### Synthetic DNA

Synthetic DNA sense (α) and antisense (β) oligonucleotides (200 nmol, lyophilized) were produced by Genomed (Warsaw, Poland). The oligonucleotides were used to obtain double-stranded DNA molecules of appropriate length with sticky ends for software input and output. An example of the construction of a DNA molecule representing the input word *abba* using sense (α) and antisense (β) oligonucleotides is described in the section ‘Construction of DNA computer elements’ below.

The oligonucleotide sequences for construction of the input molecules (input word *abba*) were *abba*(α): 5′-AATTCTAACGCGACTAATCAGCATCAGCCGACTATATTAGTTGTCATCGC-3′, and *abba*(β): 5′-GGCCGCGATGACAACTAATATAGTCGGCTGATGCTGATTAGTCGCGTTAG-3′. The oligonucleotide sequences for the detection molecule were: detect(α): 5′-AATTCGTTTATTAGTTGTCATCGC-3′ and detect(β): 5′-GGCCGCGATG-ACAACTAATAAACG-3′. The oligonucleotide sequences for the software (transition molecules) were: T122(α): 5′-AATTACTACTGTA CCCTAGTTATTAGTTGTCATCGC-3′,

T122(β): 5′-GGCCGCGATGACAACTAATAACTAGGGTACAGTAGT-3′, T162(α):

5′-AATTGAAGACGCTGATCCACGCCCTACTACTGTACCCTGGGGACCCCCCG-3′, T162(β): 5′-GGC CCGGGGGGTCCCCAGGGTACAGTAGTAGGGCGTGGATCAGCGTCTTC-3′, T107(α): 5′-AATTCTGAAG AGCTCGTTAGCTCTCTTC-3′, T107(β): 5′-GGCCGA AGAGAGCTAACGAGCTCTTCAG-3′, T24(α): 5′-AA TTGCAGCAGCTCTCATACTTTAGATTGCCTTCAG-3′, and T24(β): 5′-GGCCCTGAAGGCAATCTAAAGTATGAGAGCTGCTGC-3′.

The transition molecules, input molecule and detection molecule were prepared by annealing pairs of oligonucleotides: *abba*(α) and *abba*(β), detect(α) and detect(β), T122(α) and T122(β), T162(α) and T162(β), T107(α) and T107(β), and T24(α) and T24(β). Annealed pairs of oligonucleotides had additional sticky ends (AATT and GGCC) that enabled the insertion of DNA molecules in LITMUS 38i plasmids (see ‘Construction of DNA computer elements’).

### Enzymes

The restriction enzymes *Acu*I, *Bae*I, *Bbv*I, *Mbo*II, *Btgz*I and T4 DNA ligase were obtained from New England Biolabs (Ipswich, MA, USA). T4 polynucleotide kinase (PNK) was from Fermentas Thermo Scientific (Grand Island, NY, USA).

### Chemicals and plasmid vectors

LITMUS 38i plasmids were obtained from Fermentas Thermo Scientific. Plasmid miniprep kits and gel extraction kits were from Axygen (Union City, CA, USA). The Perfect 100 bp DNA ladder was from EurX (Gdansk, Poland). This ladder contained 13 bands with fragments sizes of 100, 200, 300, 400, 500, 600, 700, 800, 900, 1000, 1500, 2000 and 2500 bp. For easy reference, the 500 bp and 1000 bp bands are brighter than the other bands in the ladder. All other chemicals and bacterial media were from Sigma-Aldrich (St. Louis, MO, USA).

### Construction of DNA computer elements

The DNA library was constructed using LITMUS 38i plasmids as the collection of DNA molecules to represent the computer elements that had been stored and propagated in *Escherichia. coli*. Briefly, single-stranded oligonucleotides labelled according to the represented components of the automaton (the input word, detection molecule and transition molecules) were phosphorylated and annealed (by heating and slowly lowering the temperature) to form double-stranded DNA fragments. The oligonucleotide mixture was mixed with a larger fragment of LITMUS 38i plasmid digested with *Eco*RI and *Eag*I. After overnight ligation with 40 U of T4 ligase, the ligase reaction mixture was used to transform *E. coli* strain DH5-α (F– Φ80 lacZΔM15 Δ(lacZYA-argF) U169 recA1 endA1 hsdR17 (rK-, mK+) phoAsupE44 λ-thi-1 gyrA96 relA1) by the heat shock method. After DNA analysis of the colonies, the best clone was chosen and used for large scale DNA preparation with a plasmid prep kit (Axygen), according to the manufacturer’s instructions. Prior to the experiment, the appropriate automaton DNA components were obtained by PCR followed by RE digestion. We used REs to form appropriate “coding” DNA ends (*Acu*I, *Bae*I, *Btg*ZI and *Mbo*II) and *Taq* polymerase to form the second, “non-coding” (in terms of automaton) DNA ends that contained A overhangs at the 3-end. Each DNA molecule thus had one coding end that was complementary to some DNA transition molecules and one non-coding end that was incompatible with any other DNA molecules in the assay tube. This procedure eliminated the possibility of accidental, random joining of DNA automaton molecules. All molecules were purified by gel extraction prior to the experiments.

We illustrate the above scheme with a concrete example, including the method used to prepare the DNA molecule representing the input word *abba*:

**Step 1:** Sense (α) and antisense (β) oligonucleotides representing input word *abba* were placed in the assay tube.
  Sense oligonucleotide:
*abba* (α) : 5′-AATTCTAACGCGACTAATCAGCATCAGCCGACTATATTAGTTGTCATCGC-3′
    Antisense oligonucleotide:
*abba* (β) : 3′-GATTGCGCTGATTAGTCGTAGTCGGCTGATATAATCAACAGTAGCGCCGG-5′
**Step 2:** Pairs of oligonucleotides [*abba*(α) and *abba*(β)] were annealed to obtain double-stranded DNA fragments with additional sticky ends (AATT and GGCC) that enabled the insertion of DNA molecules into LITMUS 38i plasmids.
*abba* (α) : 5′-**AATT**CTAACGCGACTAATCAGCATCAGCCGACTATATTAGTTGTCATCGC-3′
*abba* (β) : 3′-GATTGCGCTGATTAGTCGTAGTCGGCTGATATAATCAACAGTAGCG**CCGG**-5′
**Step 3:** Double-stranded DNA fragments were cloned into LITMUS 38i plasmids (digested with *Eco*RI and *Eag*I).
**Step 4:** The LITMUS 38i plasmids were subsequently propagated in *E. coli.*
**Step 5:** PCR was used to obtain many copies of intermediate DNA molecules (with A overhangs at the 3-end).

**Figure:**
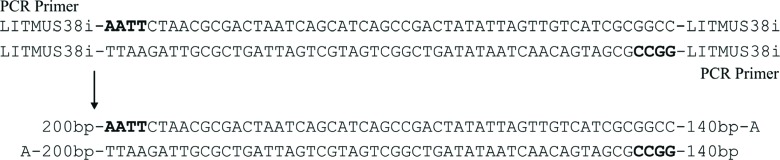
**Step 6:** The restriction enzyme (*Btgz*I) was used to form an appropriate “coding” (in relation to the automaton) DNA end.

**Figure:**
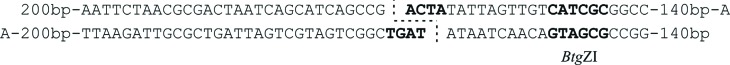

Finally, we obtained the input word *abba*:

200 bp-**AATT**CTAACGCGACTAATCAGCATCAGCCG

A-200 bp-TTAAGATTGCGCTGATTAGTCGTAGTCGGC**TGAT**

### PCR reaction

DNA molecules for the computer were obtained by PCR using a Perpetual OptiTaq PCR master mix (Eurx) in conjunction with the primers shown in Table 1. The PCR mixture (25 μL) consisted of 1.25 U of Perpetual OptiTaq DNA polymerase, 1x reaction buffer (1.5 mM MgCl_2_), 0.2 mM of each dNTP and 0.5 μM of upstream and downstream primer. The PCR conditions were as follows: initial denaturation step at 95 °C for 3 min, 30 cycles of 95 °C for 30 s, 60 °C (annealing temperature) for 30 s and 72 °C for 30 s, and a final extension step at 72°C for 5 min. PCR was done in a model PTC-100 thermal cycler (MJ Research Inc., Waltham, MA, USA). The PCR products were subsequently digested with an appropriate RE and the samples then run on 2% agarose gels and stained with ethidium bromide (0.5 μg/ml).

**Table 1:** PCR primers used in this study.

Name	Sequence (5′-3′)
Primer_2	CGTGGCTAGCGGGAAG
Primer_3	ACCATGATTACGCCAAGCTA
Primer_4	AGGAGAGCGCACGAGGGA
Primer_5	CTCACTCATTAGGCACCC
Primer_6	TGCTGCAAGGCGATTAAGTT

Transition molecules were prepared with primer_2 and primer_3 and had a final length (after digestion with RE and gel purification) of ~110 bp. The detection molecule was prepared with primer_2 and primer_4 and had a final length of 404 bp. The word molecule was prepared with primer_5 and primer_6 and had a final length of 230 bp.

### Computation reactions

Autonomous and programmable cleavage of DNA molecules by the four endonucleases was observed in one test tube. This reaction was run for 2 h in CutSmart buffer (New England Biolabs) supplemented with S-adenosylmethionine at 37 °C. The reaction tube contained a set of DNA fragments representing the input molecules, transition molecules and detection molecules, 1 U of each enzymes and 40 U of T4 DNA *ligase*. The reaction product was purified with phenol, chloroform and izoamyl alcohol (25:24:1, v/v), precipitated with ethanol and separated by electrophoresis on a 2% agarose gel. The control sample was similar to the test samples except for the absence of REs and ligase. The reactions started with ligation of the transition molecule with an input word. After the cyclic reactions of digestion followed by ligation, a final DNA fragment (the rest of the input molecule) joined to the detection molecule yielded a 614 bp DNA fragment that was detected by agarose gel electrophoresis.

## Results and Discussion

### An algorithmic method for the construction of transition molecules

The issue of how to effectively construct transition molecules in biomolecular finite automata is complex and becomes more difficult when several restriction enzymes are used. To address this problem, the paper’s first author developed an algorithm to construct transition molecules in biomolecular automata with multiple restriction enzymes, as described below.

**Figure 6:**
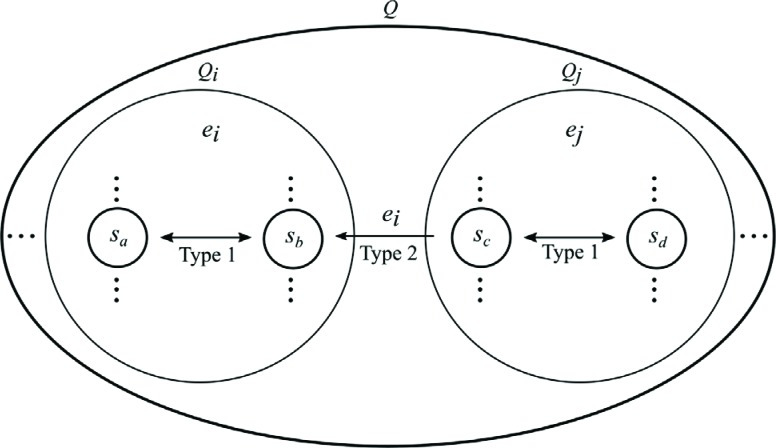
Schematic illustration of the method. The method relies on dividing the set of *Q* states of a finite automaton *M* into disjoint subsets of states and assigning only one restriction enzyme to a particular subset.

**Figure 7:**
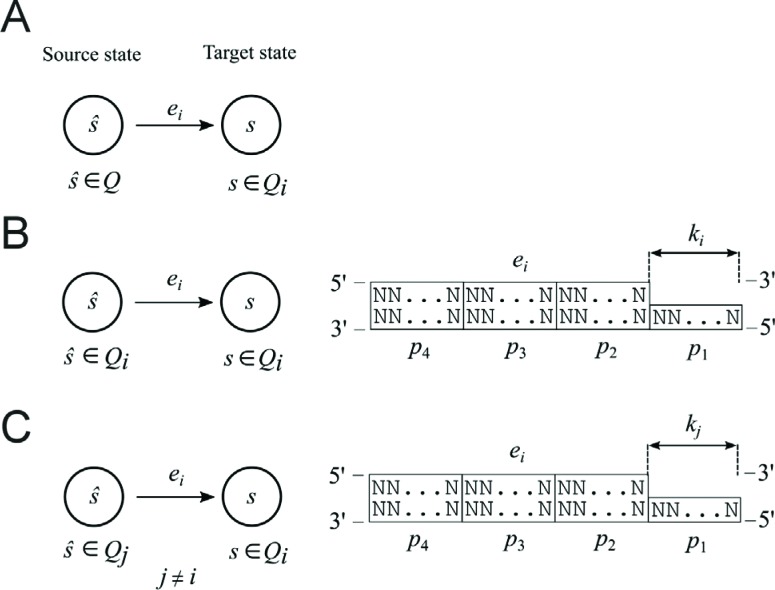
Transition rule and molecule. (A) A transition rule from the source state to the target state. (B) and (C) Construction of a type 1 and type 2 transition molecule, respectively.

The main idea of this general method relies on dividing the set of states *Q* of finite automaton *M* into disjoint subsets of states *Q*_*i*_ ⊂ *Q* (Figure 6) and assigning only one restriction enzyme *e*_*i*_ ∈ *E* (where *E=*{*e*_1_,...,*e*_*r*_} is the set of restriction enzymes) to each *Q*_*i*_ in the following way. Any transition rule with the target state *s* in *Q*_*i*_ is achieved by the enzyme *e*_*i*_. The source state may be arbitrary state *s* in *Q* (Figure 7A).

This approach generates two types of transition molecules:

**Type 1** - a transition from any state in subset *Q*_*i*_ to any state in the same subset *Q*_*i*_ is implemented by a transition molecule that satisfies the following conditions: part *p*_3_ (restriction site) of a transition molecule is characteristic of endonuclease *e*_*i*_ and part *p*_1_ (sticky end) has length *k*_*i*_ characteristic for the same endonuclease *e*_*i*_ (Figure 7B).

**Type 2** - a transition from any state in subset *Q*_*j*_ to any state of subset *Q*_*i*_, *i* ≠ *j*, is implemented by a transition molecule that satisfies the following conditions: part *p*_3_ (restriction site) of a transition molecule is characteristic of endonuclease *e*_*i*_ and part *p*_1_ (sticky end) has length *k*_*j*_ characteristic for endonuclease *e*_*j*_ (Figure 7C).

Part *p*_2_ (spacer part) of the transition molecule allows control of the depth of cutting into the input molecule and its length *l* depends on the state of the biomolecular automaton in which we want to transit after reading the next symbol of the input word. The calculation of *l* is a simple arithmetical task that involves the length of the codes in the input molecules and the distances from the restriction site in the given endonuclease. Part *p*_4_ is of fixed length *n* for all transition molecules and its length depends on biochemical reactions.

This method has an additional property that relies on the possibility of expanding the number of states in a given model of finite automata (for instance, from a six-state to a nine-state automaton). The addition of a new restriction enzyme *e*_*r*+1_, while leaving the actual transitions unchanged, allows to add new states (which form a new set *Q*_*r*+1_), and to construct new transition molecules (from states of *Q*_*r*+1_ and to states of *Q*_*r*+1_) according to two types of transition molecules: Type 1 and Type 2.

### A multistate finite automaton with multiple restriction enzymes

**Figure 8:**
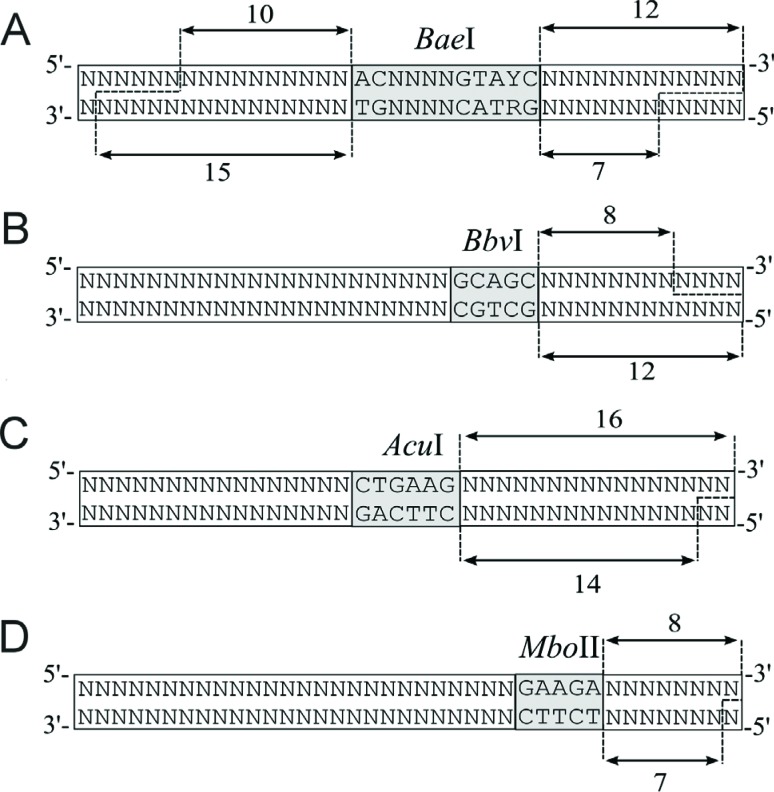
The action of restriction enzymes (A) *Bae*I, (B) *Bbv*I, (C) *Acu*I and (D) *Mbo*II. Y indicates a pyrimidine whereas R indicates a purine.

**Figure 9:**
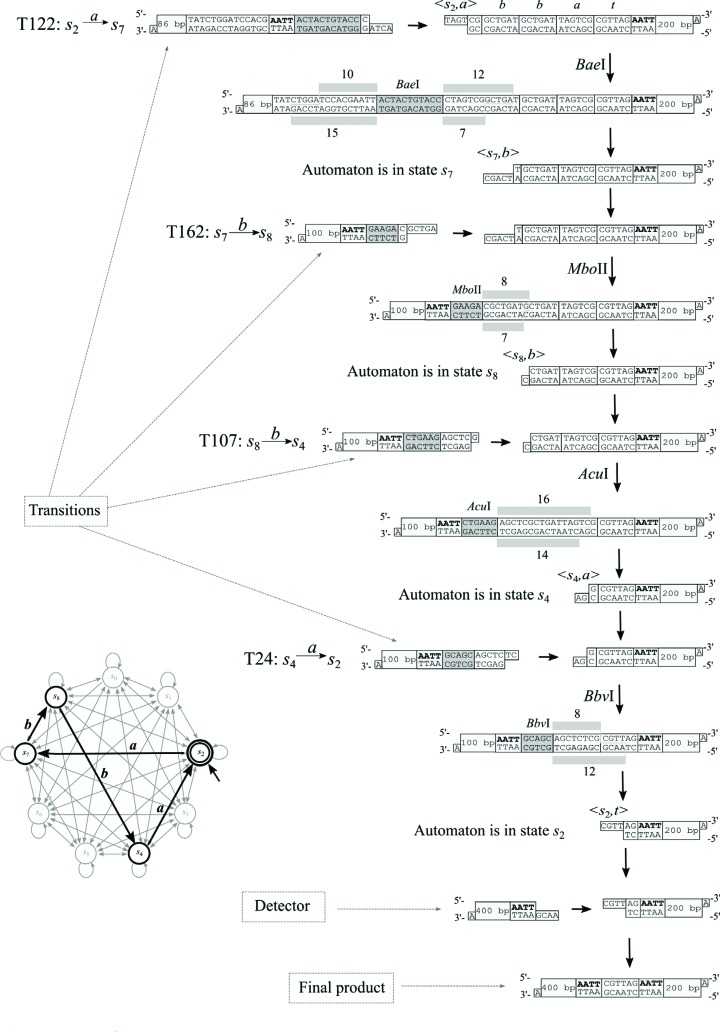
Schematic diagram of the laboratory implementation of automaton *M*_1_ using four endonucleases (*Bae*I, *Mbo*II, *Acu*I and *Bbv*I) on the word *abba*. The transition molecules allow alternating and autonomous cleavage of DNA molecules that represent the input molecule. A detector is required for recognition of the final product of computation.

The algorithmic method described above allows the construction of transition molecules for a given model of biomolecular automata by using multiple endonucleases. As the main application of this method, we decided to construct an optimal version for codes of symbols that were six base pairs in length. The model of a six-state nondeterministic finite automaton (Krasinski and Sakowski, 2008) with two endonucleases (*Bbv*I, *Acu*I) (Figure 8B,C) was extended to a nine-state nondeterministic finite automaton *M* (162 transition molecules are presented in Tables S1-S8) by including the REs *Bae*I and *Mbo*II (Figure 8A,D). These REs produce four sticky ends of lengths *k*_1_=5, *k*_2_=4, *k*_3_=2 and *k*_4_=1. The two symbols (*a* and *b*) are encoded by double-stranded DNA molecules of six base pairs in length (Figure 9). By using the procedure described by Krasinski *et al.* (2013), we could calculate the maximal number of states *p* with the formula: *p* = *n* – *k* + 1 (where *n* is the length of symbol codes and *k* is the length of the sticky ends). For example, if we use only one RE with *k*_1_=5, a maximum of two states can be achieved. To create more states (up to nine) four REs that produce four different sticky ends are required.

Using the method described here, we divided the set of nine states *Q* into four disjoint subsets of states: *Q*_1_={*s*_0_,*s*_1_,*s*_2_}, *Q*_2_={*s*_3_,*s*_4_,*s*_5_}, *Q*_3_={*s*_6_,*s*_7_} and *Q*_4_={*s*_8_}. To each subset we assigned only one restriction enzyme: *Bbv*I to subset *Q*_1_, *Acu*I to subset *Q*_2_, *Bae*I to subset *Q*_3_ and *Mbo*II to subset *Q*_4_. We distinguished two types of transition molecules: Type 1 – those with sticky ends of a length characteristic for the endonuclease that were assigned to a particular subset and Type 2 – those with sticky ends of a length not characteristic for the endonuclease that were assigned to a particular subset. All possible transition molecules for the biomolecular nondeterministic nine-state two-symbol finite automaton are shown in Supplementary material Tables S1-S8.

### Experimental assessment of the automaton with multiple restriction enzymes

**Figure 10:**
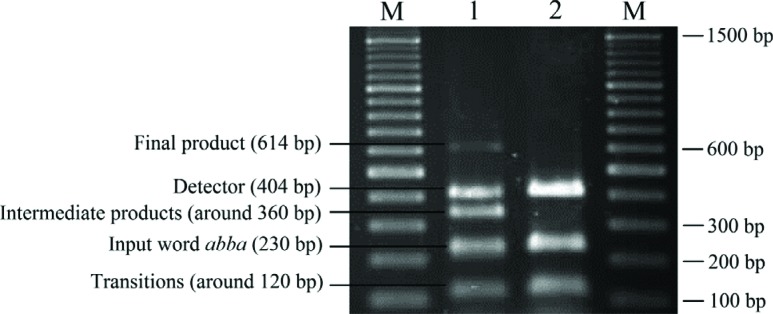
Experimental testing of automaton *M*_1_ running on the accepted word *abba*. The final product of computation was 614 bp long (see Figure 9). Abbreviations: 1 – the result of computation using four endonucleases and DNA ligase. 2 – the result of computation without the endonucleases and ligase (control experiments). M – 100 bp DNA ladder. Final product (614 bp) – DNA molecule that represented termination of the computational process in the final state *s*_2_. Detector (404 bp) – DNA molecule that recognized the final state of computation. Intermediate products (~360 bp) – intermediate DNA molecule formed during the biochemical reaction**.** Input word *abba* (230 bp) – DNA molecule that represented the word *abba*. Transitions (~120 bp) – DNA molecule that represented transition molecules.

We tested the action of automaton *M*_1_ in Figure 5B by running it on the accepted input word *abba*. These experiments focused on the key automaton element that is essential to the action of automata, namely, the autonomous and alternating action of four REs (Figure 9). If a sticky end CGTT is obtained in terminator *t* of the input word then the detection molecule will ligate to the input molecule. Since the detection molecule had no restriction sequence characteristic for any of the REs, DNA molecules 614 bp long were obtained (the previous steps produced much shorter fragments, as seen in Figures 9 and 10). Detection of the 614 bp fragment in gel electrophoresis indicated the acceptance of the input word by the automaton. The positive result of our experiment (Figure 10) proved that a multistate biomolecular automaton may act with four endonucleases. Based on this experiment, we conclude that it is possible to construct more complex finite automata using several restriction enzymes.

**Figure 11:**
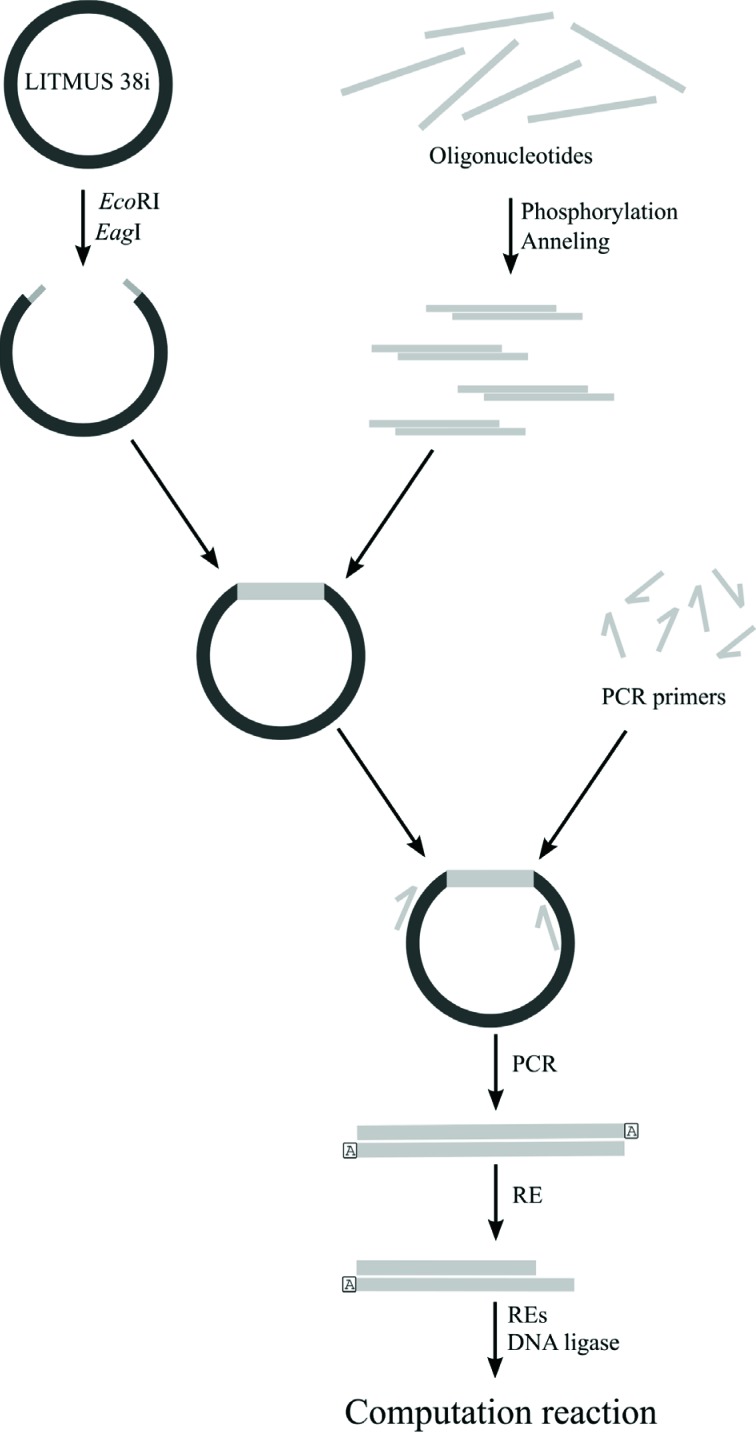
A general scheme for preparing all the automaton components (input, transition, and detection molecules).

The general scheme for preparing the automaton components differed from that of Benenson *et al.* (2001, 2003). Based on our approach, we propose to build a “DNA library”, a collection of DNA molecules representing computer elements that is stored and propagated in a population of *E. coli* through molecular cloning. Once prepared, the DNA molecules can be used at a later stage. Figure 11 summarizes the procedures for obtaining the various computer components.

## Conclusions and perspectives

The main problem with the DNA computer constructed by Ehud Shapiro’s group at the Weizmann Institute of Science was its complexity. Scaling up their DNA computer was limited by the number of states. For this reason, we focused our efforts on trying to build a more complicated DNA computer – a multistate finite automaton. Endonucleases such as *Fok*I (with four sticky ends) allow the construction of a DNA computer with at most three-states. This is a sufficient size for analysis of the five genes of small-cell lung cancer (Benenson *et al.*, 2004), although cancers are often caused by many more genes (frequently > 5). In this case, our biomolecular computer with multiple restriction enzymes could be useful for studying cancers caused by multiple genes.

The results described here show that it is possible to construct a biomolecular computer with multiple endonucleases and that this computer can act autonomously in a wet lab. Our model can be used to calculate certain algorithms, such as for vending machines that require a nine-state option for their solution automaton; this complexity cannot be dealt with using the two-state automaton described by Ehud Shapiro’s group. To a large extent, the complexity of computation with biomolecular finite automata is limited by the complexity of finite state machines that can typically only calculate simple algorithms (in polynomial time).

To prove the feasibility of our theoretical model in the wet lab we have presented the results of the laboratory implementation of a finite automaton with multiple endonucleases (*Bbv*I, *Acu*I, *Bae*I and *Mbo*II). These experiments focused on the key element essential to the action of automata, namely, the autonomous and alternating action of multiple (four) endonucleases in one test tube. One of the endonucleases (*Bae*I) cuts double-stranded DNA molecules in both directions (to the left and right). Our experiments provide a new way of using endonucleases that cut DNA molecules in both directions, thereby allowing the implementation of more powerful computational devices, e.g., pushdown automata.

The algorithmic method described here for the construction of transition molecules in biomolecular automata with multiple restriction enzymes is an *ad hoc* approach to assembling multiple restriction enzymes for the construction of biomolecular computers. This method allows the rapid construction of the main element (transition molecules) of a biomolecular finite automaton and can be used in the future to construct other computational models, e.g., pushdown automata or Turing machines made of biomolecules. An additional interesting property of this model is the possibility of increasing the number of states in the previously prepared model by adding restriction enzymes and appropriate encoding of the transition molecules. As an example of this approach, all transition molecules for a nine-state finite state automaton were encoded using commercially available restriction enzymes.

The model described here provides a basis for constructing other computational models that can be used to solve a variety of problems, such as the biomolecular Turing machines with the use of the endonuclease *Bae*I.

## Supplementary material

The following online material is available for this article:

Table S1 - Transition molecules for the subset of states *Q*_1_={*s*_0_,*s*_1_,*s*_2_} - Type 1

Table S2 - Transition molecules for the subset of states *Q*_1_={*s*_0_,*s*_1_,*s*_2_} - Type 2

Table S3 - Transition molecules for the subset of states *Q*_2_={*s*_3_,*s*_4_,*s*_5_} - Type 1

Table S4 - Transition molecules for the subset of states *Q*_2_={*s*_3_,*s*_4_,*s*_5_}- Type 2

Table S5 - Transition molecules for the subset of states *Q*_3_={*s*_6_,*s*_7_} - Type 1

Table S6 - Transition molecules for the subset of states *Q*_3_={*s*_6_,*s*_7_}- Type 2

Table S7 - Transition molecules for the subset of states *Q*_4_={*s*_8_} - Type 1

Table S8 - Transition molecules for the subset of states *Q*_4_={*s*_8_} - Type 2

## Comments to Supplementary Tables S1–S8

In all tables (Table S1 to S8), we present only important relevant parts (parts *p*_1_ and *p*_3_ – Figure 3A) of transition molecules. In practice, transition molecules were completed using fragments of the LITMUS 38i plasmid attached to the left-hand side (part *p*_4_ – Figure 3A) and by a sequence of nucleotide substitutions within block N (part *p*_2_ – Figure 3A). Each of the transition molecules contained A overhangs at the 3-end that remained after *Taq* polymerase action (PCR) used to construct each molecule. An example of a completed transition molecule from Table S1 (No. 1) is presented below.

1. Transition molecule T1 from Table S1 (No. 1, T1:):
  5′-**GCAGC**NN -3′
  3′-**CGTCG**NNCAGC-5′
2. A completed transition molecule T1 – with parts *p*_2_ and *p*_4_:
  5′- 100 bp AATT**GCAGC**CT -3′
  3′-A 100 bp TTAA**CGTCG**GACAGC-5′
