# Supplementary material for: Biomolecular computers with multiple restriction enzymes
Source: Genet Mol Biol. 2017 Oct 23;40(4):860–70. doi: 10.1590/1678-4685-GMB-2016-0132 (PMC5738618; doi:10.1590/1678-4685-GMB-2016-0132)
Supplement: Supplementary file 1 [file 1415-4757-gmb-1678-4685-GMB-2016-0132-Suppl01.pdf]

Supplementary Material to "Biomolecular computers with multiple restriction enzymes"

Table S1 - Transition molecules for the subset of states $Q_1=\{s_0,s_1,s_2\}$ - Type 1.

No.	Transition rule	Transition molecule	No.	Transition rule	Transition molecule
1	T1: $s_0 \xrightarrow{a} s_0$	5'-GCAGCNN -3'<br>3'-CGTCGNNCAGC-5'	10	T10: $s_0 \xrightarrow{b} s_0$	5'-GCAGCNN -3'<br>3'-CGTCGNNACTA-5'
2	T2: $s_0 \xrightarrow{a} s_1$	5'-GCAGCNNN -3'<br>3'-CGTCGNNNCAGC-5'	11	T11: $s_0 \xrightarrow{b} s_1$	5'-GCAGCNNN -3'<br>3'-CGTCGNNNACTA-5'
3	T3: $s_0 \xrightarrow{a} s_2$	5'-GCAGCNNNN -3'<br>3'-CGTCGNNNNCAGC-5'	12	T12: $s_0 \xrightarrow{b} s_2$	5'-GCAGCNNNN -3'<br>3'-CGTCGNNNNACTA-5'
4	T4: $s_1 \xrightarrow{a} s_0$	5'-GCAGCN -3'<br>3'-CGTCGNTCAG-5'	13	T13: $s_1 \xrightarrow{b} s_0$	5'-GCAGCN -3'<br>3'-CGTCGNGACT-5'
5	T5: $s_1 \xrightarrow{a} s_1$	5'-GCAGCNN -3'<br>3'-CGTCGNNTCAG-5'	14	T14: $s_1 \xrightarrow{b} s_1$	5'-GCAGCNN -3'<br>3'-CGTCGNNGACT-5'
6	T6: $s_1 \xrightarrow{a} s_2$	5'-GCAGCNNN -3'<br>3'-CGTCGNNNTCAG-5'	15	T15: $s_1 \xrightarrow{b} s_2$	5'-GCAGCNNN -3'<br>3'-CGTCGNNNGACT-5'
7	T7: $s_2 \xrightarrow{a} s_0$	5'-GCAGC -3'<br>3'-CGTCGATCA-5'	16	T16: $s_2 \xrightarrow{b} s_0$	5'-GCAGC -3'<br>3'-CGTCGCGAC-5'
8	T8: $s_2 \xrightarrow{a} s_1$	5'-GCAGCN -3'<br>3'-CGTCGNATCA-5'	17	T17: $s_2 \xrightarrow{b} s_1$	5'-GCAGCN -3'<br>3'-CGTCGNCGAC-5'
9	T9: $s_2 \xrightarrow{a} s_2$	5'-GCAGCNN -3'<br>3'-CGTCGNNATCA-5'	18	T18: $s_2 \xrightarrow{b} s_2$	5'-GCAGCNN -3'<br>3'-CGTCGNNCGAC-5'

N – any nucleotide (A or T, or C or G).
