# Supplementary material for: Biomolecular computers with multiple restriction enzymes
Source: Genet Mol Biol. 2017 Oct 23;40(4):860–70. doi: 10.1590/1678-4685-GMB-2016-0132 (PMC5738618; doi:10.1590/1678-4685-GMB-2016-0132)
Supplement: Supplementary file 2 [file 1415-4757-gmb-1678-4685-GMB-2016-0132-Suppl08.pdf]

Supplementary Material to “Biomolecular computers with multiple restriction enzymes”

Table S8 - Transition molecules for the subset of states $Q_4 = \{s_8\}$ - Type 2.

No.	Transition rule	Transition molecule	No.	Transition rule	Transition molecule
1	T147: $S_0 \xrightarrow{a} S_8$	5′-GAAGANNN -3′<br>3′-CTTCTNNNCAGC-5′	9	T155: $S_0 \xrightarrow{b} S_8$	5′-GAAGANNN -3′<br>3′-CTTCTNNNACTA-5′
2	T148: $S_1 \xrightarrow{a} S_8$	5′-GAAGANN -3′<br>3′-CTTCTNNTCAG-5′	10	T156: $S_1 \xrightarrow{b} S_8$	5′-GAAGANN -3′<br>3′-CTTCTNNGACT-5′
3	T149: $S_2 \xrightarrow{a} S_8$	5′-GAAGAN -3′<br>3′-CTTCTNATCA-5′	11	T157: $S_2 \xrightarrow{b} S_8$	5′-GAAGAN -3′<br>3′-CTTCTNCGAC-5′
4	T150: $S_3 \xrightarrow{a} S_8$	5′-GAAGANNNNNCG-3′<br>3′-CTTCTNNNNN -5′	12	T158: $S_3 \xrightarrow{b} S_8$	5′-GAAGANNNNNAT-3′<br>3′-CTTCTNNNNN -5′
5	T151: $S_4 \xrightarrow{a} S_8$	5′-GAAGANNNNTC-3′<br>3′-CTTCTNNNN -5′	13	T159: $S_4 \xrightarrow{b} S_8$	5′-GAAGANNNNGA-3′<br>3′-CTTCTNNNN -5′
6	T152: $S_5 \xrightarrow{a} S_8$	5′-GAAGANNNGT-3′<br>3′-CTTCTNNN -5′	14	T160: $S_5 \xrightarrow{b} S_8$	5′-GAAGANNNTG-3′<br>3′-CTTCTNNN -5′
7	T153: $S_6 \xrightarrow{a} S_8$	5′-GAAGANNAGTCG-3′<br>3′-CTTCTNN -5′	15	T161: $S_6 \xrightarrow{b} S_8$	5′-GAAGANNCTGAT-3′<br>3′-CTTCTNN -5′
8	T154: $S_7 \xrightarrow{a} S_8$	5′-GAAGANTAGTC-3′<br>3′-CTTCTN -5′	16	T162: $S_7 \xrightarrow{b} S_8$	5′-GAAGANGCTGA-3′<br>3′-CTTCTN -5′

N – any nucleotide (A or T, or C or G).
