# Supplementary material for: Biomolecular computers with multiple restriction enzymes
Source: Genet Mol Biol. 2017 Oct 23;40(4):860–70. doi: 10.1590/1678-4685-GMB-2016-0132 (PMC5738618; doi:10.1590/1678-4685-GMB-2016-0132)
Supplement: Table S2 [file 1415-4757-gmb-1678-4685-GMB-2016-0132-Suppl02.pdf]

Supplementary Material to “Biomolecular computers with multiple restriction enzymes”

Table S2 - Transition molecules for the subset of states $Q_1=\{s_0,s_1,s_2\}$ - Type 2.

No.	Transition rule	Transition molecule	No.	Transition rule	Transition molecule
1	T19: $S_3 \xrightarrow{a} S_0$	5’-GCAGCNNNNCG-3’<br>3’-CGTCGNNNN -5’	19	T37: $S_3 \xrightarrow{b} S_0$	5’-GCAGCNNNNAT-3’<br>3’-CGTCGNNNN -5’
2	T20: $S_3 \xrightarrow{a} S_1$	5’-GCAGCNNNNNCG-3’<br>3’-CGTCGNNNNN -5’	20	T38: $S_3 \xrightarrow{b} S_1$	5’-GCAGCNNNNNAT-3’<br>3’-CGTCGNNNNN -5’
3	T21: $S_3 \xrightarrow{a} S_2$	5’-GCAGCNNNNNNCG-3’<br>3’-CGTCGNNNNNN -5’	21	T39: $S_3 \xrightarrow{b} S_2$	5’-GCAGCNNNNNNAT-3’<br>3’-CGTCGNNNNNN -5’
4	T22: $S_4 \xrightarrow{a} S_0$	5’-GCAGCNNNTC-3’<br>3’-CGTCGNNN -5’	22	T40: $S_4 \xrightarrow{b} S_0$	5’-GCAGCNNNGA-3’<br>3’-CGTCGNNN -5’
5	T23: $S_4 \xrightarrow{a} S_1$	5’-GCAGCNNNNTC-3’<br>3’-CGTCGNNNN -5’	23	T41: $S_4 \xrightarrow{b} S_1$	5’-GCAGCNNNNGA-3’<br>3’-CGTCGNNNN -5’
6	T24: $S_4 \xrightarrow{a} S_2$	5’-GCAGCNNNNNTC-3’<br>3’-CGTCGNNNNN -5’	24	T42: $S_4 \xrightarrow{b} S_2$	5’-GCAGCNNNNNGA-3’<br>3’-CGTCGNNNNN -5’
7	T25: $S_5 \xrightarrow{a} S_0$	5’-GCAGCNNGT-3’<br>3’-CGTCGNN -5’	25	T43: $S_5 \xrightarrow{b} S_0$	5’-GCAGCNNTG-3’<br>3’-CGTCGNN -5’
8	T26: $S_5 \xrightarrow{a} S_1$	5’-GCAGCNNNGT-3’<br>3’-CGTCGNNN -5’	26	T44: $S_5 \xrightarrow{b} S_1$	5’-GCAGCNNNTG-3’<br>3’-CGTCGNNN -5’
9	T27: $S_5 \xrightarrow{a} S_2$	5’-GCAGCNNNNGT-3’<br>3’-CGTCGNNNN -5’	27	T45: $S_5 \xrightarrow{b} S_2$	5’-GCAGCNNNNTG-3’<br>3’-CGTCGNNNN -5’
10	T28: $S_6 \xrightarrow{a} S_0$	5’-GCAGCNAGTCG -3’<br>3’-CGTCGN -5’	28	T46: $S_6 \xrightarrow{b} S_0$	5’-GCAGCNCTGAT-3’<br>3’-CGTCGN -5’
11	T29: $S_6 \xrightarrow{a} S_1$	5’-GCAGCNNAGTCG -3’<br>3’-CGTCGNN -5’	29	T47: $S_6 \xrightarrow{b} S_1$	5’-GCAGCNNCTGAT-3’<br>3’-CGTCGNN -5’
12	T30: $S_6 \xrightarrow{a} S_2$	5’-GCAGCNNNAGTCG-3’<br>3’-CGTCGNNN -5’	30	T48: $S_6 \xrightarrow{b} S_2$	5’-GCAGCNNNCTGAT-3’<br>3’-CGTCGNNN -5’
13	T31: $S_7 \xrightarrow{a} S_0$	5’-GCAGCTAGTC-3’<br>3’-CGTCG -5’	31	T49: $S_7 \xrightarrow{b} S_0$	5’-GCAGCGCTGA-3’<br>3’-CGTCG -5’
14	T32: $S_7 \xrightarrow{a} S_1$	5’-GCAGCNTAGTC-3’<br>3’-CGTCGN -5’	32	T50: $S_7 \xrightarrow{b} S_1$	5’-GCAGCNGCTGA-3’<br>3’-CGTCGN -5’
15	T33: $S_7 \xrightarrow{a} S_2$	5’-GCAGCNNTAGTC-3’<br>3’-CGTCGNN -5’	33	T51: $S_7 \xrightarrow{b} S_2$	5’-GCAGCNNGCTGA-3’<br>3’-CGTCGNN -5’
16	T34: $S_8 \xrightarrow{a} S_0$	5’-GCAGCT-3’<br>3’-CGTCG -5’	34	T52: $S_8 \xrightarrow{b} S_0$	5’-GCAGCG-3’<br>3’-CGTCG -5’
17	T35: $S_8 \xrightarrow{a} S_1$	5’-GCAGCNT-3’<br>3’-CGTCGN -5’	35	T53: $S_8 \xrightarrow{b} S_1$	5’-GCAGCNG-3’<br>3’-CGTCGN -5’
18	T36: $S_8 \xrightarrow{a} S_2$	5’-GCAGCNNT-3’<br>3’-CGTCGNN -5’	36	T54: $S_8 \xrightarrow{b} S_2$	5’-GCAGCNNG-3’<br>3’-CGTCGNN -5’

N – any nucleotide (A or T, or C or G).
