# Supplementary material for: Biomolecular computers with multiple restriction enzymes
Source: Genet Mol Biol. 2017 Oct 23;40(4):860–70. doi: 10.1590/1678-4685-GMB-2016-0132 (PMC5738618; doi:10.1590/1678-4685-GMB-2016-0132)
Supplement: Table S3 [file 1415-4757-gmb-1678-4685-GMB-2016-0132-Suppl03.pdf]

Supplementary Material to "Biomolecular computers with multiple restriction enzymes"

Table S3 - Transition molecules for the subset of states $Q_2=\{s_3,s_4,s_5\}$ - Type 1.

No.	Transition rule	Transition molecule
1	T55: $s_3 \xrightarrow{a} s_3$	5′-CTGAAGNNNNNNNNCG-3′<br>3′-GACTTCNNNNNNNN -5′
2	T56: $s_3 \xrightarrow{a} s_4$	5′-CTGAAGNNNNNNNNNCG-3′<br>3′-GACTTCNNNNNNNNN -5′
3	T57: $s_3 \xrightarrow{a} s_5$	5′-CTGAAGNNNNNNNNNNCG-3′<br>3′-GACTTCNNNNNNNNNN -5′
4	T58: $s_4 \xrightarrow{a} s_3$	5′-CTGAAGNNNNNNNTC-3′<br>3′-GACTTCNNNNNNN -5′
5	T59: $s_4 \xrightarrow{a} s_4$	5′-CTGAAGNNNNNNNNTC-3′<br>3′-GACTTCNNNNNNNN -5′
6	T60: $s_4 \xrightarrow{a} s_5$	5′-CTGAAGNNNNNNNNNTC-3′<br>3′-GACTTCNNNNNNNNN -5′
7	T61: $s_5 \xrightarrow{a} s_3$	5′-CTGAAGNNNNNNGT-3′<br>3′-GACTTCNNNNNN -5′
8	T62: $s_5 \xrightarrow{a} s_4$	5′-CTGAAGNNNNNNNGT-3′<br>3′-GACTTCNNNNNNN -5′
9	T63: $s_5 \xrightarrow{a} s_5$	5′-CTGAAGNNNNNNNNGT-3′<br>3′-GACTTCNNNNNNNN -5′
10	T64: $s_3 \xrightarrow{b} s_3$	5′-CTGAAGNNNNNNNNAT-3′<br>3′-GACTTCNNNNNNNN -5′
11	T65: $s_3 \xrightarrow{b} s_4$	5′-CTGAAGNNNNNNNNNAT-3′<br>3′-GACTTCNNNNNNNNN -5′
12	T66: $s_3 \xrightarrow{b} s_5$	5′-CTGAAGNNNNNNNNNNAT-3′<br>3′-GACTTCNNNNNNNNNN -5′
13	T67: $s_4 \xrightarrow{b} s_3$	5′-CTGAAGNNNNNNNGA-3′<br>3′-GACTTCNNNNNNN -5′
14	T68: $s_4 \xrightarrow{b} s_4$	5′-CTGAAGNNNNNNNNGA-3′<br>3′-GACTTCNNNNNNNN -5′
15	T69: $s_4 \xrightarrow{b} s_5$	5′-CTGAAGNNNNNNNNNGA-3′<br>3′-GACTTCNNNNNNNNN -5′
16	T70: $s_5 \xrightarrow{b} s_3$	5′-CTGAAGNNNNNNTG-3′<br>3′-GACTTCNNNNNN -5′
17	T71: $s_5 \xrightarrow{b} s_4$	5′-CTGAAGNNNNNNNTG-3′<br>3′-GACTTCNNNNNNN -5′
18	T72: $s_5 \xrightarrow{b} s_5$	5′-CTGAAGNNNNNNNNTG-3′<br>3′-GACTTCNNNNNNNN -5′

N – any nucleotide (A or T, or C or G).
