# Supplementary material for: Biomolecular computers with multiple restriction enzymes
Source: Genet Mol Biol. 2017 Oct 23;40(4):860–70. doi: 10.1590/1678-4685-GMB-2016-0132 (PMC5738618; doi:10.1590/1678-4685-GMB-2016-0132)
Supplement: Table S4 [file 1415-4757-gmb-1678-4685-GMB-2016-0132-Suppl04.pdf]

Supplementary Material to “Biomolecular computers with multiple restriction enzymes”

Table S4 - Transition molecules for the subset of states $Q_2=\{s_3,s_4,s_5\}$- Type 2.

No.	Transition rule	Transition molecule	No.	Transition rule	Transition molecule
1	T73: $s_0 \xrightarrow{a} s_3$	5′-CTGAAGNNNNNN -3′<br>3′-GACTTCNNNNNNCAGC-5′	19	T91: $s_0 \xrightarrow{b} s_3$	5′-CTGAAGNNNNNN -3′<br>3′-GACTTCNNNNNNACTA-5′
2	T74: $s_0 \xrightarrow{a} s_4$	5′-CTGAAGNNNNNNN -3′<br>3′-GACTTCNNNNNNNCAGC-5′	20	T92: $s_0 \xrightarrow{b} s_4$	5′-CTGAAGNNNNNNN -3′<br>3′-GACTTCNNNNNNNACTA-5′
3	T75: $s_0 \xrightarrow{a} s_5$	5′-CTGAAGNNNNNNNN -3′<br>3′-GACTTCNNNNNNNNCAGC-5′	21	T93: $s_0 \xrightarrow{b} s_5$	5′-CTGAAGNNNNNNNN -3′<br>3′-GACTTCNNNNNNNNACTA-5′
4	T76: $s_1 \xrightarrow{a} s_3$	5′-CTGAAGNNNNN -3′<br>3′-GACTTCNNNNNTCAG-5′	22	T94: $s_1 \xrightarrow{b} s_3$	5′-CTGAAGNNNNN -3′<br>3′-GACTTCNNNNNGACT-5′
5	T77: $s_1 \xrightarrow{a} s_4$	5′-CTGAAGNNNNNN -3′<br>3′-GACTTCNNNNNNTCAG-5′	23	T95: $s_1 \xrightarrow{b} s_4$	5′-CTGAAGNNNNNN -3′<br>3′-GACTTCNNNNNNGACT-5′
6	T78: $s_1 \xrightarrow{a} s_5$	5′-CTGAAGNNNNNNN -3′<br>3′-GACTTCNNNNNNNTCAG-5′	24	T96: $s_1 \xrightarrow{b} s_5$	5′-CTGAAGNNNNNNN -3′<br>3′-GACTTCNNNNNNNGACT-5′
7	T79: $s_2 \xrightarrow{a} s_3$	5′-CTGAAGNNNN -3′<br>3′-GACTTCNNNNATCA-5′	25	T97: $s_2 \xrightarrow{b} s_3$	5′-CTGAAGNNNN -3′<br>3′-GACTTCNNNNCGAC-5′
8	T80: $s_2 \xrightarrow{a} s_4$	5′-CTGAAGNNNNN -3′<br>3′-GACTTCNNNNNATCA-5′	26	T98: $s_2 \xrightarrow{b} s_4$	5′-CTGAAGNNNNN -3′<br>3′-GACTTCNNNNNCGAC-5′
9	T81: $s_2 \xrightarrow{a} s_5$	5′-CTGAAGNNNNNN -3′<br>3′-GACTTCNNNNNNATCA-5′	27	T99: $s_2 \xrightarrow{b} s_5$	5′-CTGAAGNNNNNN -3′<br>3′-GACTTCNNNNNNCGAC-5′
10	T82: $s_6 \xrightarrow{a} s_3$	5′-CTGAAGNNNNNAGTCG-3′<br>3′-GACTTCNNNNN -5′	28	T100: $s_6 \xrightarrow{b} s_3$	5′-CTGAAGNNNNNCTGAT-3′<br>3′-GACTTCNNNNN -5′
11	T83: $s_6 \xrightarrow{a} s_4$	5′-CTGAAGNNNNNNAGTCG-3′<br>3′-GACTTCNNNNNN -5′	29	T101: $s_6 \xrightarrow{b} s_4$	5′-CTGAAGNNNNNNCTGAT-3′<br>3′-GACTTCNNNNNN -5′
12	T84: $s_6 \xrightarrow{a} s_5$	5′-CTGAAGNNNNNNNAGTCG-3′<br>3′-GACTTCNNNNNNN -5′	30	T102: $s_6 \xrightarrow{b} s_5$	5′-CTGAAGNNNNNNNCTGAT-3′<br>3′-GACTTCNNNNNNN -5′
13	T85: $s_7 \xrightarrow{a} s_3$	5′-CTGAAGNNNNTAGTC-3′<br>3′-GACTTCNNNN -5′	31	T103: $s_7 \xrightarrow{b} s_3$	5′-CTGAAGNNNNGCTGA-3′<br>3′-GACTTCNNNN -5′
14	T86: $s_7 \xrightarrow{a} s_4$	5′-CTGAAGNNNNNTAGTC-3′<br>3′-GACTTCNNNNN -5′	32	T104: $s_7 \xrightarrow{b} s_4$	5′-CTGAAGNNNNNGCTGA-3′<br>3′-GACTTCNNNNN -5′
15	T87: $s_7 \xrightarrow{a} s_5$	5′-CTGAAGNNNNNNTAGTC-3′<br>3′-GACTTCNNNNNN -5′	33	T105: $s_7 \xrightarrow{b} s_5$	5′-CTGAAGNNNNNNGCTGA-3′<br>3′-GACTTCNNNNNN -5′
16	T88: $s_8 \xrightarrow{a} s_3$	5′-CTGAAGNNNNT-3′<br>3′-GACTTCNNNN -5′	34	T106: $s_8 \xrightarrow{b} s_3$	5′-CTGAAGNNNNG-3′<br>3′-GACTTCNNNN -5′
17	T89: $s_8 \xrightarrow{a} s_4$	5′-CTGAAGNNNNNT-3′<br>3′-GACTTCNNNNN -5′	35	T107: $s_8 \xrightarrow{b} s_4$	5′-CTGAAGNNNNNG-3′<br>3′-GACTTCNNNNN -5′
18	T90: $s_8 \xrightarrow{a} s_5$	5′-CTGAAGNNNNNNT-3′<br>3′-GACTTCNNNNNN -5′	36	T108: $s_8 \xrightarrow{b} s_5$	5′-CTGAAGNNNNNNG-3′<br>3′-GACTTCNNNNNN -5′

N – any nucleotide (A or T, or C or G).
