# Supplementary material for: Biomolecular computers with multiple restriction enzymes
Source: Genet Mol Biol. 2017 Oct 23;40(4):860–70. doi: 10.1590/1678-4685-GMB-2016-0132 (PMC5738618; doi:10.1590/1678-4685-GMB-2016-0132)
Supplement: Table S5 [file 1415-4757-gmb-1678-4685-GMB-2016-0132-Suppl05.pdf]

Supplementary Material to "Biomolecular computers with multiple restriction enzymes"

Table S5 - Transition molecules for the subset of states $Q_3=\{s_6,s_7\}$ - Type 1.

No.	Transition rule	Transition molecule	No.	Transition rule	Transition molecule
1	T109: $s_6 \xrightarrow{a} s_6$	5′-ACNNNNGTAYCNAGTCG-3′<br>3′-TGNNNNCATRGN -5′	5	T113: $s_6 \xrightarrow{b} s_6$	5′-ACNNNNGTAYCNCTGAT-3′<br>3′-TGNNNNCATRGN -5′
2	T110: $s_6 \xrightarrow{a} s_7$	5′-ACNNNNGTAYCNNAGTCG-3′<br>3′-TGNNNNCATRGNN -5′	6	T114: $s_6 \xrightarrow{b} s_7$	5′-ACNNNNGTAYCNNCTGAT-3′<br>3′-TGNNNNCATRGNN -5′
3	T111: $s_7 \xrightarrow{a} s_7$	5′-ACNNNNGTAYCNTAGTC-3′<br>3′-TGNNNNCATRGN -5′	7	T115: $s_7 \xrightarrow{b} s_7$	5′-ACNNNNGTAYCNGCTGA-3′<br>3′-TGNNNNCATRGN -5′
4	T112: $s_7 \xrightarrow{a} s_6$	5′-ACNNNNGTAYCTAGTC-3′<br>3′-TGNNNNCATRG -5′	8	T116: $s_7 \xrightarrow{b} s_6$	5′-ACNNNNGTAYCGCTGA-3′<br>3′-TGNNNNCATRG -5′

N – any nucleotide (A or T, or C or G), R = A or G, Y = C or T.
