# Supplementary material for: Biomolecular computers with multiple restriction enzymes
Source: Genet Mol Biol. 2017 Oct 23;40(4):860–70. doi: 10.1590/1678-4685-GMB-2016-0132 (PMC5738618; doi:10.1590/1678-4685-GMB-2016-0132)
Supplement: Table S6 [file 1415-4757-gmb-1678-4685-GMB-2016-0132-Suppl06.pdf]

# Supplementary Material to "Biomolecular computers with multiple restriction enzymes"

**Table S6 -** Transition molecules for the subset of states $Q_3=\{s_6,s_7\}$- Type 2.

| No. | Transition rule | Transition molecule | No. | Transition rule | Transition molecule |
|---|---|---|---|---|---|
| 1 | T117: $S_0 \xrightarrow{a} S_6$ | 5′-ACNNNNGTAYCNN -3′<br>3′-TGNNNNCATRGNNCAGC-5′ | 15 | T131: $S_0 \xrightarrow{b} S_6$ | 5′-ACNNNNGTAYCNN -3′<br>3′-TGNNNNCATRGNNACTA-5′ |
| 2 | T118: $S_0 \xrightarrow{a} S_7$ | 5′-ACNNNNGTAYCNNN -3′<br>3′-TGNNNNCATRGNNNCAGC-5′ | 16 | T132: $S_0 \xrightarrow{b} S_7$ | 5′-ACNNNNGTAYCNNN -3′<br>3′-TGNNNNCATRGNNNACTA-5′ |
| 3 | T119: $S_1 \xrightarrow{a} S_6$ | 5′-ACNNNNGTAYCN -3′<br>3′-TGNNNNCATRGNTCAG-5′ | 17 | T133: $S_1 \xrightarrow{b} S_6$ | 5′-ACNNNNGTAYCN -3′<br>3′-TGNNNNCATRGNGACT-5′ |
| 4 | T120: $S_1 \xrightarrow{a} S_7$ | 5′-ACNNNNGTAYCNN -3′<br>3′-TGNNNNCATRGNNTCAG-5′ | 18 | T134: $S_1 \xrightarrow{b} S_7$ | 5′-ACNNNNGTAYCNN -3′<br>3′-TGNNNNCATRGNNGACT-5′ |
| 5 | T121: $S_2 \xrightarrow{a} S_6$ | 5′-ACNNNNGTAYC -3′<br>3′-TGNNNNCATRGATCA-5′ | 19 | T135: $S_2 \xrightarrow{b} S_6$ | 5′-ACNNNNGTAYC -3′<br>3′-TGNNNNCATRGCGAC-5′ |
| 6 | T122: $S_2 \xrightarrow{a} S_7$ | 5′-ACNNNNGTAYCN -3′<br>3′-TGNNNNCATRGNATCA-5′ | 20 | T136: $S_2 \xrightarrow{b} S_7$ | 5′-ACNNNNGTAYCN -3′<br>3′-TGNNNNCATRGNCGAC-5′ |
| 7 | T123: $S_3 \xrightarrow{a} S_6$ | 5′-ACNNNNGTAYCNNNNCG-3′<br>3′-TGNNNNCATRGNNNN -5′ | 21 | T137: $S_3 \xrightarrow{b} S_6$ | 5′-ACNNNNGTAYCNNNNAT-3′<br>3′-TGNNNNCATRGNNNN -5′ |
| 8 | T124: $S_3 \xrightarrow{a} S_7$ | 5′-ACNNNNGTAYCNNNNNCG-3′<br>3′-TGNNNNCATRGNNNNN -5′ | 22 | T138: $S_3 \xrightarrow{b} S_7$ | 5′-ACNNNNGTAYCNNNNNAT-3′<br>3′-TGNNNNCATRGNNNNN -5′ |
| 9 | T125: $S_4 \xrightarrow{a} S_6$ | 5′-ACNNNNGTAYCNNNTC-3′<br>3′-TGNNNNCATRGNNN -5′ | 23 | T139: $S_4 \xrightarrow{b} S_6$ | 5′-ACNNNNGTAYCNNNGA-3′<br>3′-TGNNNNCATRGNNN -5′ |
| 10 | T126: $S_4 \xrightarrow{a} S_7$ | 5′-ACNNNNGTAYCNNNNTC-3′<br>3′-TGNNNNCATRGNNNN -5′ | 24 | T140: $S_4 \xrightarrow{b} S_7$ | 5′-ACNNNNGTAYCNNNNGA-3′<br>3′-TGNNNNCATRGNNNN -5′ |
| 11 | T127: $S_5 \xrightarrow{a} S_6$ | 5′-ACNNNNGTAYCNNGT-3′<br>3′-TGNNNNCATRGNN -5′ | 25 | T141: $S_5 \xrightarrow{b} S_6$ | 5′-ACNNNNGTAYCNNTG-3′<br>3′-TGNNNNCATRGNN -5′ |
| 12 | T128: $S_5 \xrightarrow{a} S_7$ | 5′-ACNNNNGTAYCNNNGT-3′<br>3′-TGNNNNCATRGNNN -5′ | 26 | T142: $S_5 \xrightarrow{b} S_7$ | 5′-ACNNNNGTAYCNNNTG-3′<br>3′-TGNNNNCATRGNNN -5′ |
| 13 | T129: $S_8 \xrightarrow{a} S_6$ | 5′-ACNNNNGTAYCT-3′<br>3′-TGNNNNCATRG -5′ | 27 | T143: $S_8 \xrightarrow{b} S_6$ | 5′-ACNNNNGTAYCG-3′<br>3′-TGNNNNCATRG -5′ |
| 14 | T130: $S_8 \xrightarrow{a} S_7$ | 5′-ACNNNNGTAYCNT-3′<br>3′-TGNNNNCATRGN -5′ | 28 | T144: $S_8 \xrightarrow{b} S_7$ | 5′-ACNNNNGTAYCNG-3′<br>3′-TGNNNNCATRGN -5′ |

N – any nucleotide (A or T, or C or G), R = A or G, Y = C or T.
