# Supplementary material for: Biomolecular computers with multiple restriction enzymes
Source: Genet Mol Biol. 2017 Oct 23;40(4):860–70. doi: 10.1590/1678-4685-GMB-2016-0132 (PMC5738618; doi:10.1590/1678-4685-GMB-2016-0132)
Supplement: Table S7 [file 1415-4757-gmb-1678-4685-GMB-2016-0132-Suppl07.pdf]

Supplementary Material to "Biomolecular computers with multiple restriction enzymes"

Table S7 - Transition molecules for the subset of states $Q_4=\{s_8\}$ - Type 1.

No.	Transition rule	Transition molecule	No.	Transition rule	Transition molecule
1	T145: $s_8 \xrightarrow{a} s_8$	5′-GAAGANT-3′<br>3′-CTTCTN -5′	2	T146: $s_8 \xrightarrow{b} s_8$	5′-GAAGANG-3′<br>3′-CTTCTN -5′

N – any nucleotide (A or T, or C or G).
